# Effects of Intravenous Dexmedetomidine on Emergence Agitation in Children under Sevoflurane Anesthesia: A Meta-Analysis of Randomized Controlled Trials

**DOI:** 10.1371/journal.pone.0099718

**Published:** 2014-06-16

**Authors:** Chengliang Zhang, Jiajia Hu, Xinyao Liu, Jianqin Yan

**Affiliations:** 1 Department of Anesthesiology, Xiangya Hospital, Central South University, Changsha, China; 2 Department of Cardiology, The Third Xiangya Hospital of Central South University, Changsha, China; San Raffaele Scientific Institute, Italy

## Abstract

**Objective:**

Emergence agitation (EA) is a common complication in children under sevoflurane anesthesia. The aim of this meta-analysis was to evaluate the effects of intravenous dexmedetomidine on EA in children under sevoflurane anesthesia.

**Methods:**

A comprehensive literature search was conducted to identify clinical trials that evaluated the effects of intravenous dexmedetomidine and placebo on EA in children under sevoflurane anesthesia. The search collected trials from MEDLINE, Cochrane Central Register of Controlled Trials (CENTRAL), Embase, and Web of Science. Analysis was conducted using STATA version 12.0. Data from each trial were pooled using relative ratio (RR) for dichotomous data or weighted mean difference (WMD) for continuous data and corresponding 95% confidence interval (95% CI). Heterogeneity assessment, sensitivity analysis, and publication bias were performed.

**Results:**

Twelve trials, in which 459 patients received dexmedetomidine and 353 patients received placebo, were included in this analysis. We found that intravenous dexmedetomidine decreased the incidences of EA (RR = 0.346, 95% CI 0.263 to 0.453, P<0.001), and postoperative pain (RR = 0.405, 95% CI 0.253 to 0.649, P<0.001). Intravenous dexmedetomidine also prolonged extubation time (WMD = 0.617, 95% CI 0.276 to 958, P<0.001), and emergence time (WMD = 0.997, 95% CI 0.392 to 1.561, P = 0.001). Further evidences are required to evaluate the incidence of postoperative nausea and vomiting (PONV). Sensitivity analysis strengthened evidences for lower incidences of EA, pain, and prolonged extubation time, and emergence time. Funnel plots did not detect any significant publication bias.

**Conclusion:**

Meta-analysis demonstrated that dexmedetomidine decreased the incidence of EA in children under sevoflurane anesthesia.

## Introduction

Sevoflurane is a widely used inhalational anesthetic for pediatric anesthesia because of its low pungency, low blood–gas partition coefficient, rapid onset, fast recovery properties, minimal cardiac depressive effect, and low toxicity [1], [2]. However, sevoflurane anesthesia is associated with a high incidence (10%–80%) of emergence agitation (EA) in children [3]–[6]. The etiology of EA derives from numerous factors including rapid awakening, pain, preoperative anxiety, surgery type, personality, and anesthetic administered. EA is also associated with complications such as self-injury, anxiety, and increased costs for additional medical care.

Drugs such as the α2-adrenoceptor agonist dexmedetomidine may improve EA after sevoflurane anesthesia. Dexmedetomidine is highly specific for the α2-adrenoceptor and has an 8-fold higher affinity than clonidine [7]. It has sedative, analgesic, and anxiolytic properties with few adverse effects [8]. Several clinical trials have shown that intravenous dexmedetomidine significantly reduces the incidence of EA in children under sevoflurane anesthesia [9]–[11]. To evaluate effects of intravenous dexmedetomidine on emergence agitation, pain, postoperative nausea and vomiting (PONV), extubation time, PACU length of stay and emergence time in children under sevoflurane anesthesia, compared with placebo from randomized trials, we performed this meta-analysis.

## Methods

### Ethics

No ethics approval was required.

### Protocol

The study protocol followed the recommendations of the PRISMA statement and Cochrane Collaboration for systematic reviews and meta-analysis [12], [13].

### Search strategy and selection of included studies

A comprehensive literature search for published randomized controlled trials was conducted. High-sensitivity and low-specificity search principles were used in PubMed, Embase, Cochrane Central Register of Controlled Trials (CENTRAL) and Web of Science without language restriction by two reviewers in duplicate. The keywords “agitation”, “delirium”, “children”, “infant”, “sevoflurane”, “dexmedetomidine,” and their alternative words were combined by the Boolean meanings of “AND” (for “agitation”, “children”, “sevoflurane”, “dexmedetomidine”) and “OR” (among alternative words). The last electronic search was performed in 15 March 2014. We also searched the references from the eligible articles or textbooks to find potential sources. If the full text could not be found, authors were contacted to provide a copy of the original article.

Clinical trials comparing dexmedetomidine and placebo (saline or lactated Ringer's solution) intravenously administered perioperatively to prevent EA in children (age 1–14 years) under standardized anesthesia protocols with sevoflurane were included in analysis. We excluded trials that combined administered 2 prophylactic agents in 1 group during operation. We also excluded data from scientific meetings, correspondence, case reports, reviews, and animal studies. We evaluated quality of included trials using the Cochrane Collaboration's tool for assessing risk of bias in randomized trials [14]. There are seven items to assess random sequence generation: allocation concealment, blinding of participants and personnel, blinding of outcome assessment, incomplete outcome data, selective reporting, and other bias using high, low or unclear risk of bias [15].

### Data extraction

Two authors independently reviewed the inclusion criteria of all retrieved articles. Two independent authors assessed the study quality and extracted the data. For each study, the following data were collected: first author, publication year, patient age, surgery type, ASA classification, number of patients, control group, intervention group, sevoflurane anesthesia protocol, the incidence of EA, the incidence of postoperative nausea and vomiting (PONV), and postoperative pain, extubation time, postanesthesia care unit (PACU) length of stay, and emergence time. All disagreements were resolved by consensus through discussion among authors and the final decision was made by the corresponding author.

### Statistical analysis

Analysis was conducted using STATA version 12.0. We compared relative ratios (RR) for dichotomous data or weighted mean differences (WMD) for continuous data with corresponding 95% confidence intervals (95% CI) for each trial. RR<1 indicated that the incidence of the test target in the dexmedetomidine group was lower than that in the placebo group. Each analysis was assessed for statistical heterogeneity using the Cochran's Q test and I^2^ test. P<0.10 was considered significant. If P>0.10 and I^2^<50%, the fixed effects model was used; otherwise the random effects model was used. Sensitivity analysis was conducted by removing each study individually to assess the quality and consistency of the results. Begg's funnel plots and Egger's linear regression test were used to detect potential publication bias. An asymmetric funnel plot indicated the presence of publication bias, whereas a symmetric plot suggested that there was no publication bias.

## Results

### Literature Search Findings

**Figure 1:**
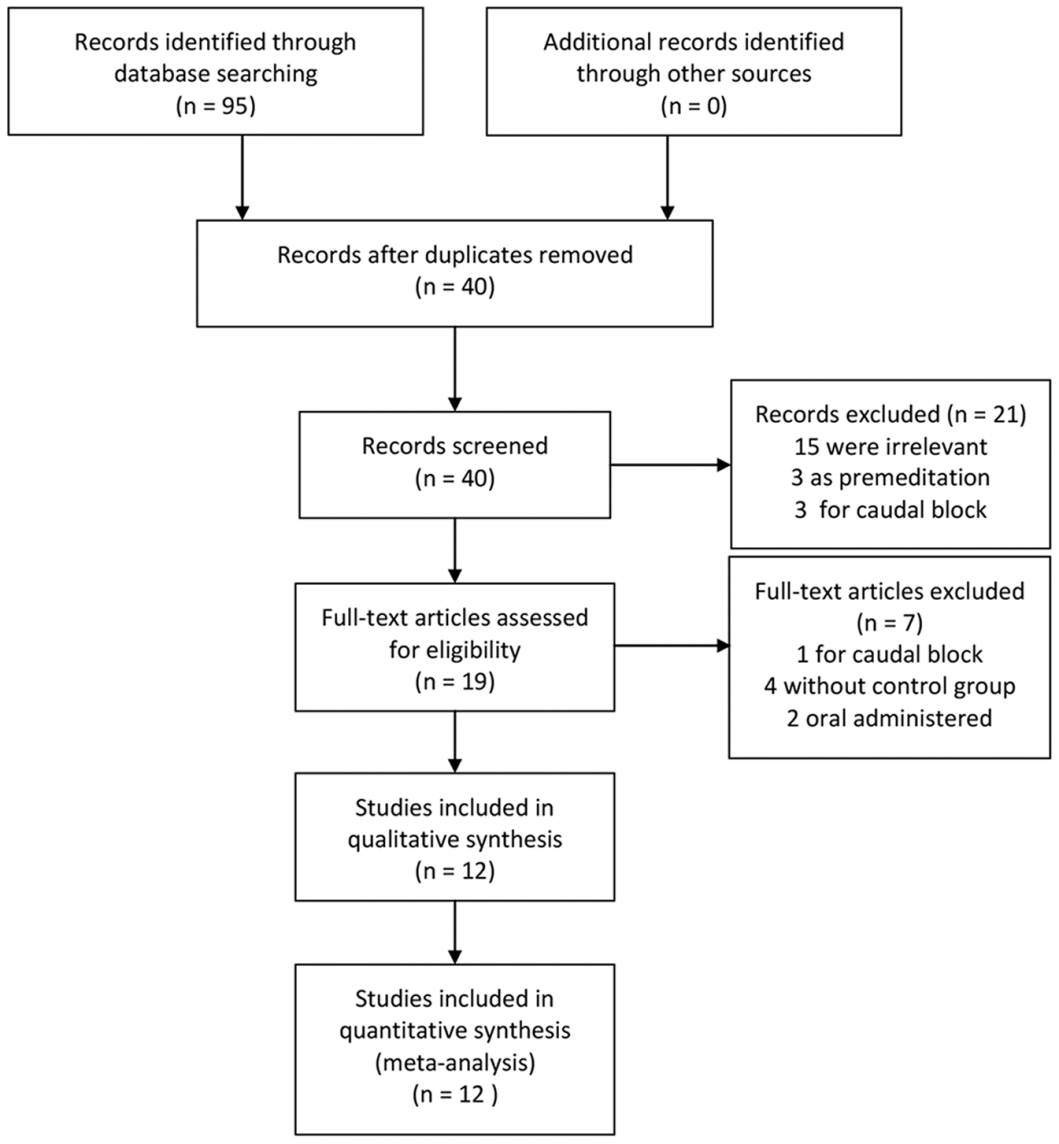
Flow chart of meta-analysis.

A total of 67 trials were identified with 55 excluded by the inclusion criteria. The remaining 12 relevant trials included 459 patients who received dexmedetomidine and 353 patients who received the placebo. Details of the selection process are summarized in Figure 1. Dexmedetomidine was administered by single dose in 9 trials [9]–[11], [16]–[21], continuous infusion in 3 trials [22]–[24]. The placebo included saline in 11 trials [9]–[11], [16]–[22], [24] and lactated Ringer's solution in 1 trial [23]. There were 2 different dexmedetomidine doses examined in 3 trials [10], [19], [23]. For trials that comparison between control group and multiple intervention groups using different dexmedetomidine dose, we combined intervention groups to create a single pair-wise comparison. For dichotomous outcomes, both the sample sizes and the numbers of people with events were summed across groups. For continuous outcomes, means and standard deviations were combined using a formula recommended by the handbook [25]. The characteristics of included articles are listed in Table 1. The risk of bias assessment showed that the quality of included trials was high (Table 2). All meta-analysis results were showed in table 3.

**Table 1:** Characteristics of included trials.

Author Year	Age(years)	Surgery	Study/Control	Study Intervention	Pre-medication	Sevoflurane induction	Sevoflurane maintain	Assessment Methods of EA
Ibacache [10] 2004	1–10	Inguinal hernia repair, orchiopexy, or circumcision	60/30	Single dose dexmedetomidine 0.15 ug/kg (0.3 ug/kg)IV	No	8% sevoflurane and 50% N_2_O in O_2_	3% sevoflurane in 50% N_2_O	4-point EA scale >2
Shukry [24] 2005	1–10	Outpatient surgical procedures	23/23	Dexmedetomidine in a concentration of 0.2 ug/(kg*h) IV	No	8% sevoflurane in O_2_	sevoflurane to achieve a BIS 40–60	4-point EA scale >2
Guler [11] 2005	3∼7	Adenotonsillectomy	30/30	Dexmedetomidine 0.5 ug/kg IV before the end of the surgery	Acetaminophen15 mg/kg (oral)	8% sevoflurane and 50% N_2_O in O_2_	1.5–2% sevoflurane in 60% N_2_O and 40% O_2_	5-point Behavior Scale >3
Isik [9] 2006	1.510	MRI examination (LMA)	21/21	Dexmedetomidine 1 ug/kg IV over 2 min after induction	No	8% sevoflurane in 2.5 L/min N_2_O and 2.5 L/min O_2_	1.5% sevoflurane in 2 L/min N_2_O and 2 L/min O_2_	5-point Behavior scaleOf >3
Erdil [18] 2009	2–7	Adenoidectomy	30/30	Dexmedetomidine 0.5 mg/kg IV.	40 mg/kg paracetamol (rectally)	50% N_2_O and 8% sevoflurane in O_2_	sevoflurane 1.5 to 2.5% (inspired concentration) in 70% N_2_O/O_2_	5-point Behavior scale Of >3
Sato [21] 2010	1–9	Ambulatory surgery	39/41	Dexmedetomidine 0.3 ug/kg IV over 10 min	No	8% sevoflurane in 6 L/min O_2_	2%–5% sevoflurane in 2 L/min O_2_ and 4 L/min air	4-point EA scale >2
Meng [23] 2012	5–14	Tonsillectomy	80/40	Dexmedetomidine 0.5 (1.0) mg/kg IV over10 min, maintained with 0.2(0.4) mg/(kg*h) over the surgery	40 ug/kg midazolam (IV)	None	1.5%–2.5% sevoflurane fresh O_2_ gas flow of 2.0 L/min	4-point EA scale >2
Xu [20] 2012	3–7	Vitreoretinal surgery	30/30	Dexmedetomidine 0.5 ug/kg IV over a period of 10 min	No	8% sevoflurane in O_2_	Sevoflurane (1%–2% end-tidal concentration) in O_2_	4-point EA scale >2
Gupta [22] 2013	8–12	Corrective spinal dysraphism	18/18	Dexmedetomidine 1 mg/kg bolus over 10 min followed by 0.5 mg/(kg*h)	0.2 mg glycopyrrolate (intramuscular)	Sevoflurane 8%,	60% N_2_O in O_2_ and sevoflurane at a fresh gas flow of 3 L/min	5-point Agitation Cole score >3
Chen [17] 2013s	2–7	Strabismus surgery(LMA)	27/24	Dexmedetomidine 1 ug/kg IV in the surgery	No	8% sevoflurane in 5 L/min O_2_ (FiO_2_ = 1.0)	8% sevoflurane in 5 L/min O_2_ (FiO_2_ = 1.0)	20-point Pediatric AnesthesiaEmergence Delirium ≥10
Ali [16] 2013	2–6	Adenotonsillectomy	40/40	Dexmedetomidine 0.3 ug/kg IV 5 min before the end of surgery	0.5 mg/kg midazolam (oral)	8% sevoflurane and 70% N_2_O in O_2_	2%–3% sevoflurane, 60% N_2_O in O_2_	5-point Aonos scale >2
He [19] 2013	3–7	Minor surface surgery (LMA)	61/26	Dexmedetomidine 0.5 ug/kg (1 ug/kg) IV for 10 min during surgery	No	8%sevoflurane in O_2_	sevoflurane in O_2_ (1 L/min) and air (1 L/min)	5-point Behavior scale Of >3

**Table 2:** Risk of bias assessment for evaluation the quality of each included trials.

Year	study	Random sequence generation	Allocation concealment	Blinding of participants and personnel	Blinding of outcome assessment	Incomplete outcome data	Selective reporting	Other bias
2004	Ibacache [10]	Low	Unclear	Low	Low	Low	Low	Low
2005	Shukry [24]	Low	Unclear	Low	Low	Unclear	Low	Unclear
2005	Guler [11]	Low	Unclear	Low	Low	Low	Low	Unclear
2006	Isik [9]	Low	Unclear	Low	Low	Low	Low	Low
2009	Erdil [18]	Low	Low	Low	Low	Low	Low	Low
2010	Sato [21]	Low	Unclear	Low	Low	Low	Low	Unclear
2012	Meng [23]	Low	Unclear	Low	Low	Low	Low	Unclear
2012	Xu [20]	Low	Low	Low	Low	Low	Low	Low
2013	Gupta [22]	Low	Unclear	Low	Low	Low	Low	Low
2013	Chen [17]	Low	Unclear	Low	Low	Unclear	Low	Low
2013	Ali [16]	Low	Low	Low	Low	Low	Low	Low
2013	He [19]	Low	Unclear	Low	Low	Low	Low	Low

**Table 3:** Meta-analysis results of all items.

Items	Trials	I-square	P for heterogeneity	Model	RR/WMD	95% CI	P	Begg	Egger
EA	12	0.00%	0.666	Fixed	0.346	(0.263,0.453)	0.000	0.115	0.11
PONV	7	0.00%	0.622	Fixed	0.593	(0.391,0.901)	0.014	0.764	0.922
pain	5	0.00%	0.879	Fixed	0.405	(0.253,0.649)	0.000	0.221	0.304
Extubation time	9	31.30%	0.168	Fixed	0.617	(0.276,0.958)	0.000	0.917	0.961
PACU length of stay	3	0.00%	0.898	Fixed	4.597	(−0.080,9.275)	0.054	0.296	0.388
Emergence time	8	0.00%	0.574	Fixed	0.977	(0.392,1.561)	0.001	0.266	0.346

### EA incidence

**Figure 2:**
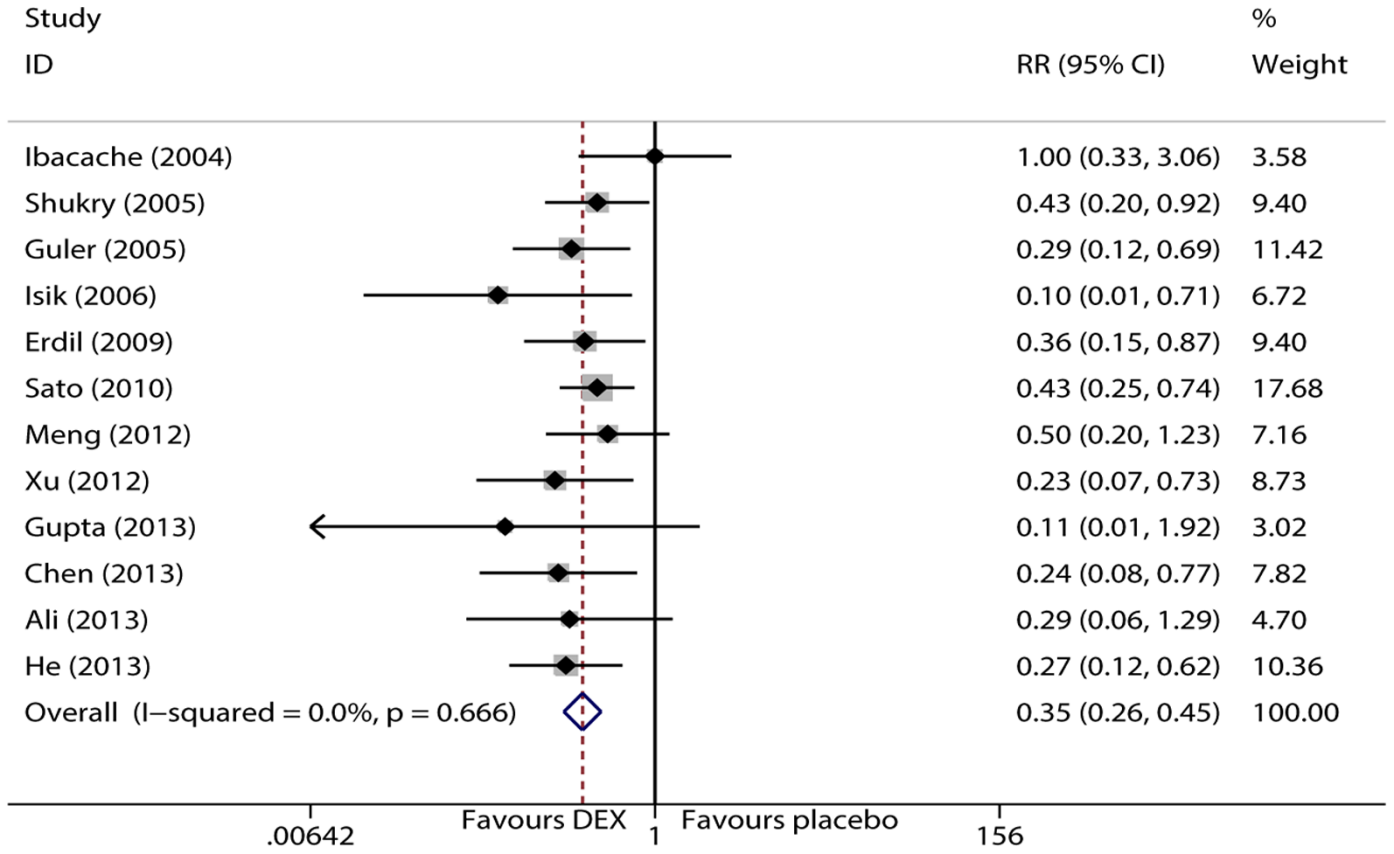
Forest plot of EA incidence.

**Figure 3:**
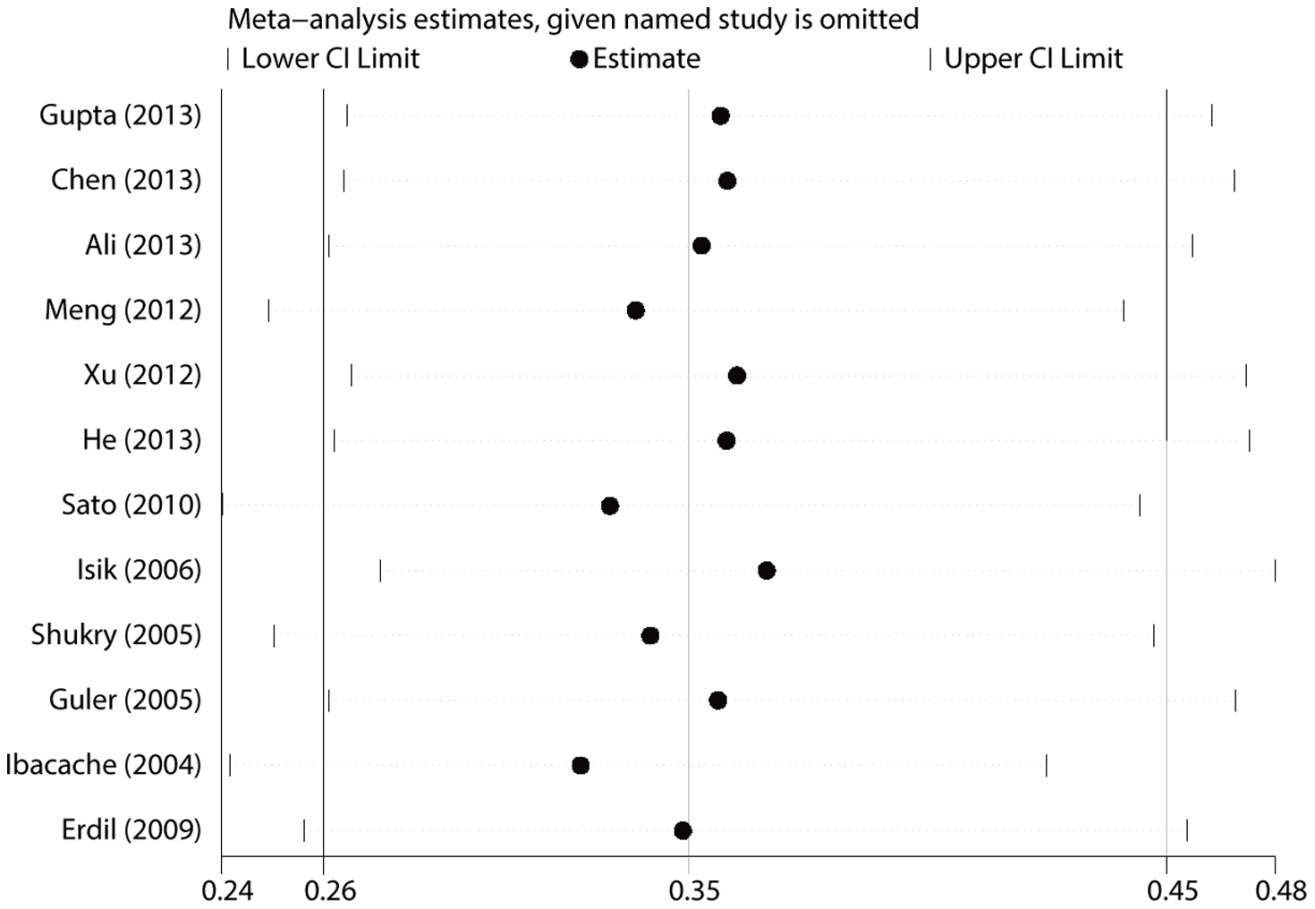
Sensitivity analysis result of EA incidence.

**Figure 4:**
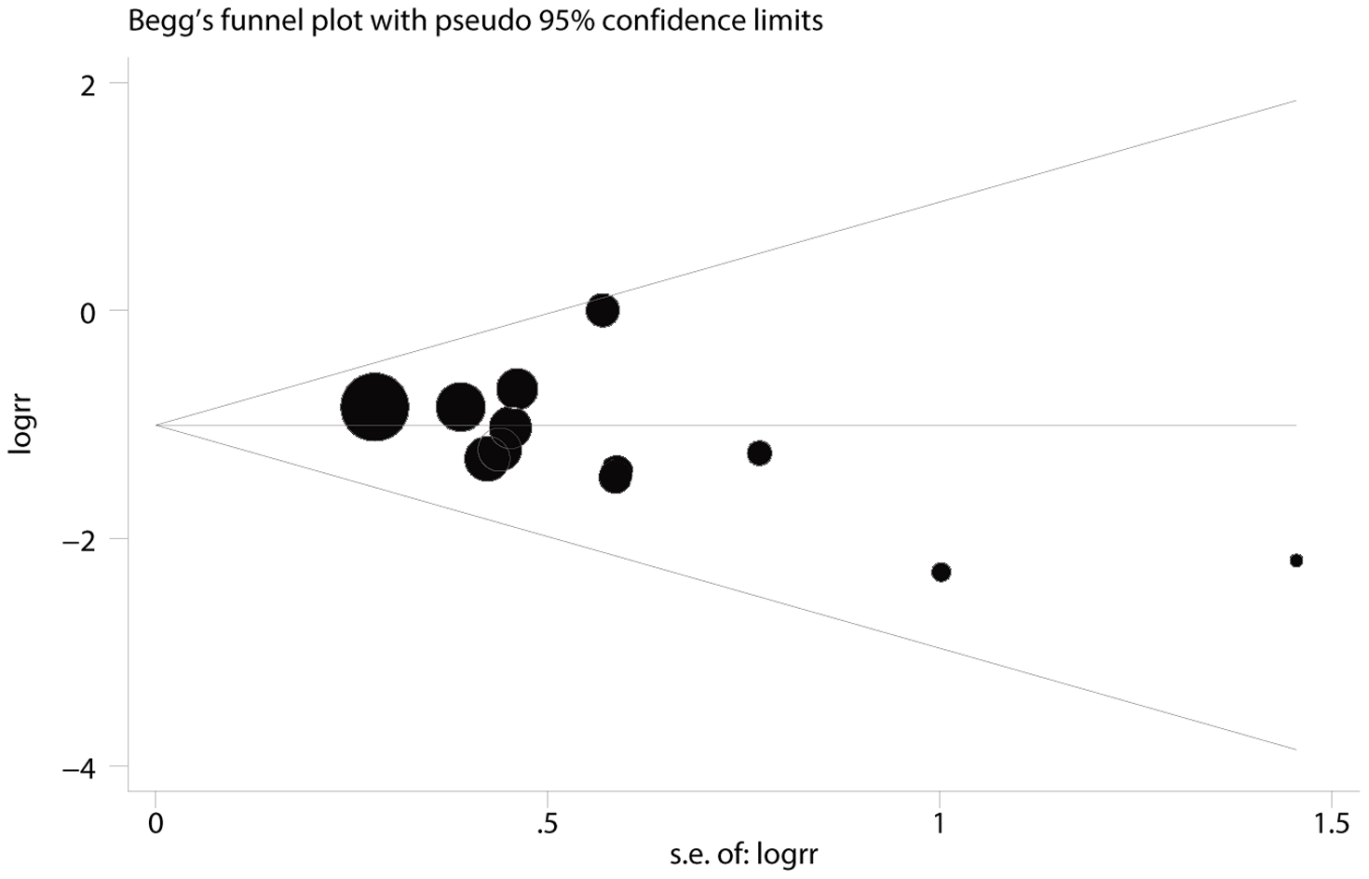
Funnel plot of EA incidence.

EA was assessed using a 5-point scale of Agitation Cole score (ACS), Behavior Scale or Pediatric Anesthesia Emergence Delirium (PAED) scale. There were 12 trials [9]–[11], [16]–[24] that examined the incidence of EA in children under sevoflurane anesthesia. No statistically significant heterogeneity was observed according to the I^2^and Q tests (I^2^<0.1%, P = 0.666), and therefore, the fixed effects model was selected. The pooled result showed that dexmedetomidine significantly decreased the incidence of EA in children under sevoflurane anesthesia (RR = 0.346, 95% CI 0.263 to 0.453, P<0.001, Figure 2). The result was stable when sensitivity analysis that involved removing 1 trial once from the pooled result was conducted (RR_min_ = 0.321, 95% CI_min_ 0.242 to 0.426, and RR_max_ = 0.363, 95% CI_max_ 0.276 to 0.478, Figure 3). The Begg's funnel plots (P = 0.115) and Egger's linear regression test (P = 0.110) indicated the probability of publication bias was low (Figure 4).

### PONV incidence

**Figure 5:**
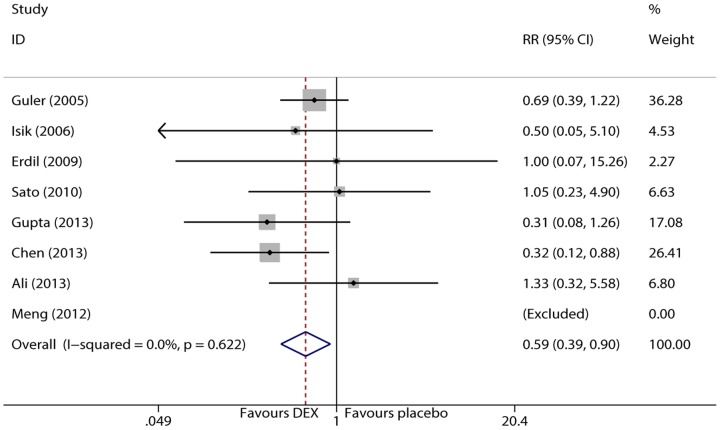
Forest plot of PONV incidence.

PONV is assessed by nausea and vomiting behaviors from the entrance of PACU to 24 hr.after surgery. 7 trials [9], [11], [16], [17], [21]–[23] examined the incidence of PONV in children under sevoflurane anesthesia. According to the I^2^ and Q tests, there was no statistically significant heterogeneity (I^2^<0.1%, P = 0.622), and therefore, the fixed effects model was selected. The pooled result showed that dexmedetomidine significantly decreased the incidence of PONV in children under sevoflurane anesthesia (RR = 0.593, 95% CI 0.391 to 0.901, P = 0.014, Figure 5). However, when the trial of Gupta et al [22] or Chen et al [17] was removed from the pooled trials, a CI of 1 was generated in the 95% CI (0.421 to 1.009 or 0.433 to 1.099 respectively). This decreased the reliability of the test, and therefore, further evidences are required to reach a clear conclusion.

### Pain incidence in PACU

**Figure 6:**
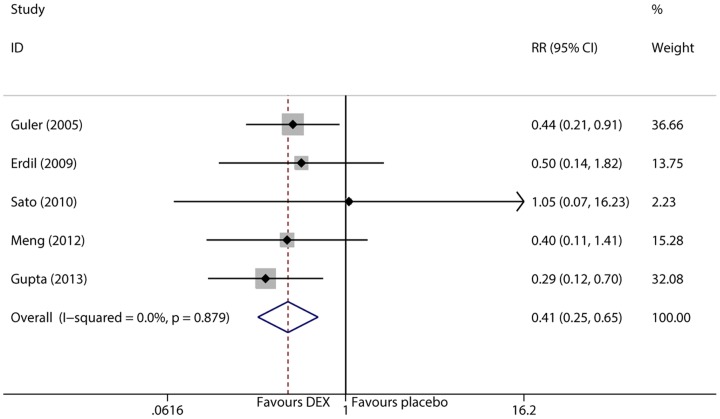
Forest plot of pain incidence.

Postoperative pain in PACU was assessed by visual analog scale (VAS) or Objective Pain Scale (OPS) during the period in PACU and for 24 hr on the ward. There were 5 trials [11], [18], [21]–[23] examined the incidence of pain in PACU. Data were homogeneous according to the I^2^ and Q tests (I^2^<0.1%, P = 0.879), and therefore, the fixed effects model was selected. The pooled result showed that dexmedetomidine significantly decreased the incidence of pain in children in PACU. (RR = 0.405, 95% CI 0.253 to 0.649, P<0.001, Figure 6). Removal of individual trials did not significantly alter the result. Funnel plots did not display significant asymmetry.

### Extubation time

**Figure 7:**
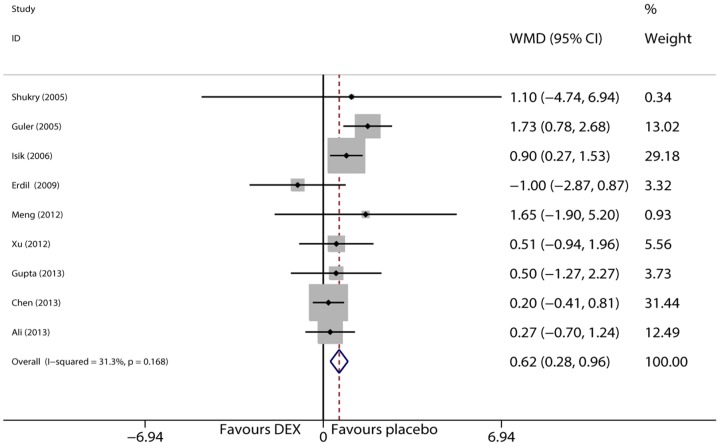
Forest plot of extubation time.

Extubation time which was measured as the time interval between anesthetic discontinuation and extubation was examined in 9 trials [9], [11], [16]–[18], [20], [22]–[24]. Data were homogeneous (I^2^ = 31.3%, P = 0.168). The combined result from the fixed effects model suggested that dexmedetomidine prolonged extubation time (WMD  = 0.617 min, 95% CI 0.276 to 0.958, P<0.001, Figure 7). Sensitivity analysis was conducted to examine the influence of each trial on the overall risk estimate and the results were stable.

### PACU length of stay

**Figure 8:**
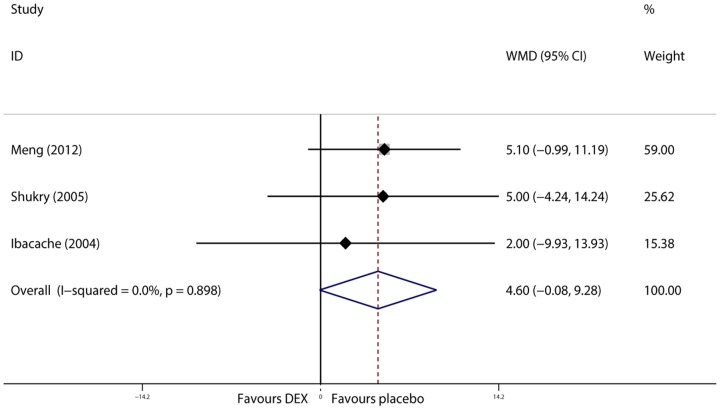
Forest plot of PACU length of stay.

PACU length of stay was examined in 3 trials [10], [23], [24]. We selected the fixed effects model to pool data because data was homogeneous (I^2^<0.1%, P = 0.898). We found that PACU length of stay in the dexmedetomidine group was prolonged compared to that in the placebo group (WMD = 4.597 min, 95% CI −0.080 to 9.275, P = 0.054, Figure 8). Sensitivity analysis revealed that the results were stable when trials were removed one by one.

### Emergence time

**Figure 9:**
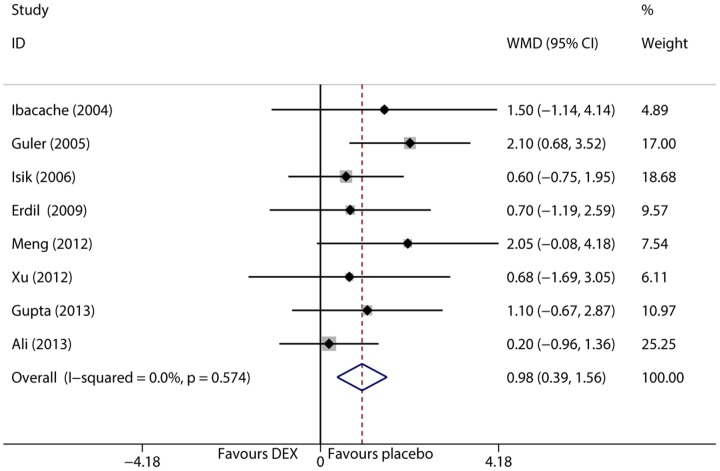
Forest plot of emergence time.

Emergence time was defined as the time from discontinuation of the anesthetic to opening of eyes and was examined in 8 trials [9]–[11], [16], [18], [20], [22], [23]. The I^2^ test and Q tests showed that data was homogeneous (I^2^<0.1%, P = 0.574), and therefore, the fixed effect model was selected. The pooled result demonstrated that dexmedetomidine prolonged emergence time (WMD = 0.977 min, 95% CI 0.392 to 1.561, P = 0.001, Figure 9). Sensitivity analysis showed that the pooled result was not influenced by individual trials.

### Adverse effects

There were no serious adverse events such as oxygen desaturation, hypotension, bradycardia, or postoperative respiratory depression in any patient at any time during the study period, except 3children had bronchospasm in the control group [9].

## Discussion

The early stages of EA in children are characterized by crying, excitation, agitation, and delirium [1]. Sevoflurane is associated with a high incidence of EA, and there is a general agreement amongst anesthetists that sevoflurane can increase the incidence of EA in the recovery stage in children compared to propofol [3]–[5]. Meta-analysis confirmed that EA occurs more frequently in children under sevoflurane anesthesia than under propofol anesthesia [26]. In addition, another meta-analysis demonstrated that EA occurs more frequently under sevoflurane anesthesia than under halothane anesthesia [5]. The reported incidence of EA following sevoflurane anesthesia varies from 10%–80% [6]. The etiology of EA includes rapid awakening, pain, preoperative anxiety, personality, surgery type, and anesthetic [5]. Furthermore, children between the age of 2 and 5 years are more likely to suffer from EA [27]. EA has additional complications in pediatric patients that include an increased risk of self-injury, dissatisfaction, and associated extra medical care [21].

A previous meta-analysis showed that the α2-adrenoceptor agonists dexmedetomidine and clonidine were effective in preventing EA related to sevoflurane and desflurane in children [8]. It is difficult to clear which is more effective. Thus, we only focused on the effects of a single agent—dexmedetomidine which may prevent EA in children under sevoflurane anethesia. Our meta-analysis suggests that dexmedetomidine can significantly reduce the incidence of EA after emergence from sevoflurane anesthesia in pediatric patients. These results also support dexmedetomidine as an effective and safe agent in preventing EA.

Some authors insist that rapid awakening is the cause of EA [28]. The low blood–gas solubility and rapid recovery characteristics of sevoflurane may contribute to EA [29]–[34]. In a meta-analysis of Kanaya et al [26] showed that the incidence of EA is higher under sevoflurane anesthesia than that under propofol anesthesia in children, extubation time in propofol group was slightly longer (WMD = 1.09 min, 95% CI 0.096 to 2.09), however, because of the significant data heterogeneity, it is difficult to confirm whether rapid emergence plays a role in the higher incidence of EA after sevoflurane anesthesia. In our findings that children administered dexmedetomidine had slightly prolonged extubation time, and emergence time (WMD = 0.617 min, 95% CI 0.276 to 0.958, and WMD = 0.997 min, 95% CI 0.392 to 1.561 respectively), and lower incidence of EA. However, the prolonged time is slight without clinically significant. Thus, it is difficult to confirm that rapid emergence is a contributing factor to EA.

Pain is considered to be one of the major causes of EA. However, symptoms of screaming, irritability, and anxiety potentially associated with pain are very difficult to distinguish from those of EA, especially in young children. Some studies suggest that EA can be provoked without pain. Isik et al [9] reported that EA was observed in 48% of pediatric patients under sevoflurane anesthesia when undergoing magnetic resonance imaging. Several studies [26], [31] demonstrate that children under propofol anethesia, which does not have analgesia effects, had lower incidence of EA. In addition, children recovered smoothly and pleasantly compared with those under sevoflurane anethesia [26], [31]. Others argue that using fentanyl as a preemptive analgesic can reduce the incidence of EA without delaying emergence associated with desflurane or sevoflurane anesthesia in children [31], [32], [35]–[37]. From the results of our meta-analysis, children who administered dexmedetomidine had lower incidence of EA, as well as frequency of postoperative pain. Thus, we believe that pain may play a role in the incidence of EA in children.

Dexmedetomidine, a highly specific a2-adrenoceptor agonist with sedative, analgesic, and anxiolytic properties without significant respiratory depression at clinical dosages, has been widely used in pediatric and adult populations [7], [8], [38]. Our findings support several prospective clinical trials in children that dexmedetomidine significantly reduces the incidence of EA after sevoflurane anesthesia [9]–[11]. In addition, we found that dexmedetomidine prolonged emergence time and extubation time. Dexmedetomidine is generally well tolerated with few adverse effects. It has little effect on direct memory impairment, respiratory depression, opioid-related pruritus, and PONV at clinical doses [39]. Numerous studies demonstrate that dexmedetomidine has an opioid-sparing effect [40]–[46] which can contribute to sufficient analgesia duration, emergence stage, and improve appropriate sedation to offset rapid elimination. The combined actions of attenuated pain, prolonged sedative duration and depth also reduce the incidence of EA. Dexmedetomidine infusions are generally well tolerated with few adverse effects [47], [48]. In all the included trials, we did not find any serious adverse effects. We propose that the sedative and analgesic properties of dexmedetomidine work together to reduce the incidence of EA. Thus, dexmedetomidine appears to be a promising agent to prevent EA in children under sevoflurane anesthesia.

Our meta-analysis has a number of limitations. First, each study was based on a different study protocol (including the administration methods of dexmedetomidine and sevoflurane) that may cause significant data heterogeneity, although, based on our data analysis at least, we did not find significant heterogeneity. Second, the age range of children differed between the trials examined, with the symptoms of EA being more likely from 2 to 5 years [27]. In our study, age ranged from 1.5 to 14 years, and this large range may influence the incidence of EA.

## Conclusions

Our meta-analysis demonstrated that dexmedetomidine decreases the incidence of EA in children under sevoflurane anesthesia. Our analysis also indicated that dexmedetomidine can decrease the incidence of postoperative pain, prolong emergence time, and extubation time. These findings are reinforced by our sensitivity and publication bias analyses. However, more studies are required to evaluate the effect of dexmedetomidine on the prevention of PONV. We propose that dexmedetomidine is a promising agent to prevent EA in children under sevoflurane anesthesia.

## Supporting Information

Checklist S1 **PRISMA 2009 Checklist.**
(DOC)
