# Supplementary material for: Effects of Intravenous Dexmedetomidine on Emergence Agitation in Children under Sevoflurane Anesthesia: A Meta-Analysis of Randomized Controlled Trials
Source: PLoS One. 2014 Jun 16;9(6):e99718. doi: 10.1371/journal.pone.0099718 (PMC4059696; doi:10.1371/journal.pone.0099718)
Supplement: Checklist S1 — PRISMA 2009 Checklist. (DOC) [file pone.0099718.s001.doc]

| **Section/topic** | **#** | **Checklist item** | **Reported on page #** |
| --- | --- | --- | --- |
| **TITLE** | | |  |
| Title | 1 | Effects of intravenous dexmedetomidine on emergence agitation in children under sevoflurane anesthesia:  A meta-analysis of randomized controlled trials | Effects of intravenous dexmedetomidine on emergence agitation in children under sevoflurane anesthesia |
| **ABSTRACT** | | |  |
| Structured summary | 2 | Objective: Emergence agitation (EA) is a common complication in children under sevoflurane anesthesia. The aim of this meta-analysis was to evaluate the effects of intravenous dexmedetomidine on EA in children under sevoflurane anesthesia.  Methods: A comprehensive literature search was conducted to identify clinical trials that evaluated the effects of intravenous dexmedetomidine and placebo on EA in children under sevoflurane anesthesia. The search collected trials from MEDLINE, Cochrane Central Register of Controlled Trials (CENTRAL), Embase, and Web of Science. Analysis was conducted using STATA version 12.0. Data from each trial were pooled using relative ratios (RR) for dichotomous data or weighted mean differences (WMD) for continuous data and corresponding 95% confidence intervals (95%CI). Heterogeneity assessment, sensitivity analysis, and publication bias were performed.  Results: Twelve trials, in which 459 patients received dexmedetomidine and 353 patients received placebo, were included in this analysis. We found that intravenous dexmedetomidine decreased the incidences of EA (RR = 0.346, 95%CI 0.263 to 0.453, P < 0.001) and postoperative pain (RR = 0.405, 95%CI 0.253 to 0.649, P < 0.001). Intravenous dexmedetomidine also prolonged extubation time (WMD = 0.617, 95%CI 0.276 to 958, P < 0.001), and emergence time (WMD = 0.997, 95%CI 0.392 to 1.561, P = 0.001). Further evidences are required to evaluate the incidences of postoperative nausea and vomiting (PONV) and postanesthesia care unit (PACU) length of stay. Sensitivity analysis strengthened evidence for lower incidences of EA and pain and prolonged extubation time, and emergence time. Funnel plots did not detect any significant publication bias.  Conclusion: Meta-analysis demonstrated that dexmedetomidine decreased the incidence of EA in children under sevoflurane anesthesia. | Abstract |
| **INTRODUCTION** | | |  |
| Rationale | 3 | Sevoflurane is a widely used inhalational anesthetic for pediatric anesthesia. However, sevoflurane anesthesia is associated with a high incidence (10%–80%) of emergence agitation (EA) in children. The etiology of EA derives from numerous factors including rapid awakening, pain, preoperative anxiety, surgery type, personality, and anesthetic administered. EA is also associated with complications such as self-injury, anxiety, and increased costs for additional medical care. Several prospective clinical trials showed that dexmedetomidine significantly reduces the incidence of EA in children recovering from sevoflurane anesthesia. Our meta-analysis was aimed to systematically evaluate the effect of dexmedetomidine on preventing emergence agitation, pain, postoperative nausea and vomiting (PONV), and analysis the the etiology of EA. | Introduction |
| Objectives | 4 | To evaluate effects of intravenous dexmedetomidine on emergence agitation, pain, postoperative nausea and vomiting (PONV), extubation time, PACU length of stay and emergence time in children under sevoflurane anesthesia, compared with placebo from randomized trials, we performed this meta-analysis. | Introduction |
| **METHODS** | | |  |
| Protocol and registration | 5 | No protocol and registration for this meta analysis. | Protocol |
| Eligibility criteria | 6 | Clinical trials comparing dexmedetomidine and placebo (saline or lactated Ringer’s solution) administered perioperatively to prevent EA in children (age 1–14 years) under standardized anesthesia protocols with sevoflurane were included in analysis. | Search strategy and selection of included studies |
| Information sources | 7 | A comprehensive literature search for published randomized controlled trials was conducted. High-sensitivity and low-specificity search principles were used in PubMed, Embase, Cochrane Central Register of Controlled Trials (CENTRAL) and Web of Science without language destriction by two reviewers in duplicate. The keywords “agitation”, “delirium”, “children”, “infant” , “sevoflurane”, “dexmedetomidine,” and their alternative words were combined by the Boolean meanings of “AND” ( for “agitation”, “children”, “sevoflurane”, “dexmedetomidine”) and “OR” (among alternative words). The last electronic search was performed in 15 March 2014. We also searched the references from the eligible articles or textbooks to find potential sources. If the full text could not be found, authors were contacted to provide a copy of the original article. | Search strategy and selection of included studies |
| Search | 8 | Search strategy for PUBMED:  ((((((((((((((child) OR children) OR infant) OR infants) OR pediatric) OR pediatrics) OR child[MeSH Terms])))) AND ((((sevoflurane) OR "sevoflurane"[Supplementary Concept])))) AND ((((agitation) OR delirium) OR behavior) OR "Delirium"[Mesh])) AND (((((((((("α2-adrenoceptor agonist") OR "α2-adrenoceptor agonists") OR "alpha2 adrenoceptor agonist") OR "alpha2 adrenoceptors agonist") OR "α2-AR agonist") OR "α2-AR agonists") OR "alpha2 AR agonist") OR "alpha2 AR agonists")) OR ((Dexmedetomidine) OR "Dexmedetomidine"[Mesh]))) AND (((((((((((((((((((((("randomized controlled trial"[Publication Type]) OR "controlled clinical trial"[Publication Type]) OR "randomized controlled trials"[MeSH Terms]) OR "random allocation"[MeSH Terms]) OR "double-blind method"[MeSH Terms]) OR "single-blind method"[MeSH Terms]) OR "clinical trial"[Publication Type]) OR "clinical trials"[MeSH Terms]) OR "clinical trial"[Text Word]) OR "singl*"[Text Word]) OR "doubl*"[Text Word]) OR "trebl*"[Text Word]) OR "tripl*"[Text Word])) AND (("mask*"[Text Word]) OR "blind"[Text Word]))) OR ((((((((((("Latin square"[Text Word]) OR "placebos"[Text Word]) OR "placebo*"[Text Word]) OR "random*"[Text Word]) OR "follow-up studies"[MeSH Terms]) OR "prospective studies"[MeSH Terms]) OR "cross-over studies"[MeSH Terms]) OR "control*"[Text Word]) OR "prospective*"[Text Word]) OR "volunteer*"[Text Word]) OR "research design"[MeSH Major Topic]))) NOT (("Animals"[Mesh]) NOT "Humans"[Mesh]))) NOT ((((("comment"[Publication Type]) OR "editorial"[Publication Type]) OR "meta-analysis"[Publication Type]) OR "practice- guideline"[Publication Type]) OR "review"[Publication Type])))) AND ((("random*") OR "randomized controlled trial") OR ("Randomized Controlled Trial" [Publication Type] OR "Randomized Controlled Trials as Topic"[Mesh] OR "Controlled Clinical Trial" [Publication Type])) | Search strategy and selection of included studies |
| Study selection | 9 | Retrieved articles were reviewed for inclusion by one author, and criteria for inclusion were independently checked by 2 authors. First, we excluded the duplicate studies, then screened the titles, abstracts, and full text, at last chose the trials that match the inclusion criteria. | Data extraction |
| Data collection process | 10 | Two authors independently extracted all the relevant information from original reports. Another two authors checked all extracted data. When any necessary outcomes were not reported, we contacted the authors to request them to provide the accurate data. If this was unsuccessful, we computed it when data sufficient. We selected only one study from the duplicate studies. | Data extraction |
| Data items | 11 | For each study, the following data were collected: first author, publication year, patient age, surgery type, ASA classification, number of patients, control group, intervention group, sevoflurane anesthesia protocol, the incidences of EA, incidence postoperative nausea and vomiting (PONV), and postoperative pain, extubation time, postanesthesia care unit (PACU) duration, and emergence time. | Data extraction |
| Risk of bias in individual studies | 12 | We evaluated quality of included trials using the Cochrane Collaboration’s tool for assessing risk of bias in randomized trials. There are seven items to assess random sequence generation, allocation concealment, blinding of participants and personnel, blinding of outcome assessment, incomplete outcome data, selective reporting, and other bias using high, low or unclear risk of bias. | Search strategy and selection of included studies |
| Summary measures | 13 | We compared relative ratios (RR) for dichotomous data or weighted mean differences (WMD) for continuous data with corresponding 95% confidence intervals (95%CI) for each trial. | Statistical analysis |
| Synthesis of results | 14 | Each analysis was assessed for statistical heterogeneity using the Cochran’s Q test and I^2^ test. P < 0.10 was considered significant. If P > 0.10 and I^2^ < 50%, the fixed effects model was used; otherwise the random effects model was used. | Statistical analysis |

| **Section/topic** | **#** | **Checklist item** | **Reported on page #** |
| --- | --- | --- | --- |
| Risk of bias across studies | 15 | Begger's funnel plots and Egger's linear regression test were used to detect the publication bias. We recognized the selective reporting by comparison the integrity of the data. | Statistical analysis |
| Additional analyses | 16 | Sensitivity analysis was conducted by removing each study individually to assess the quality and consistency of the results. | Statistical analysis |
| **RESULTS** | | |  |
| Study selection | 17 | A total of 67 trials were identified with 55 excluded by the inclusion criteria. The remaining 12 relevant trials included 459 patients who received dexmedetomidine and 353 patients who received the placebo. Details of the selection process are summarized as below  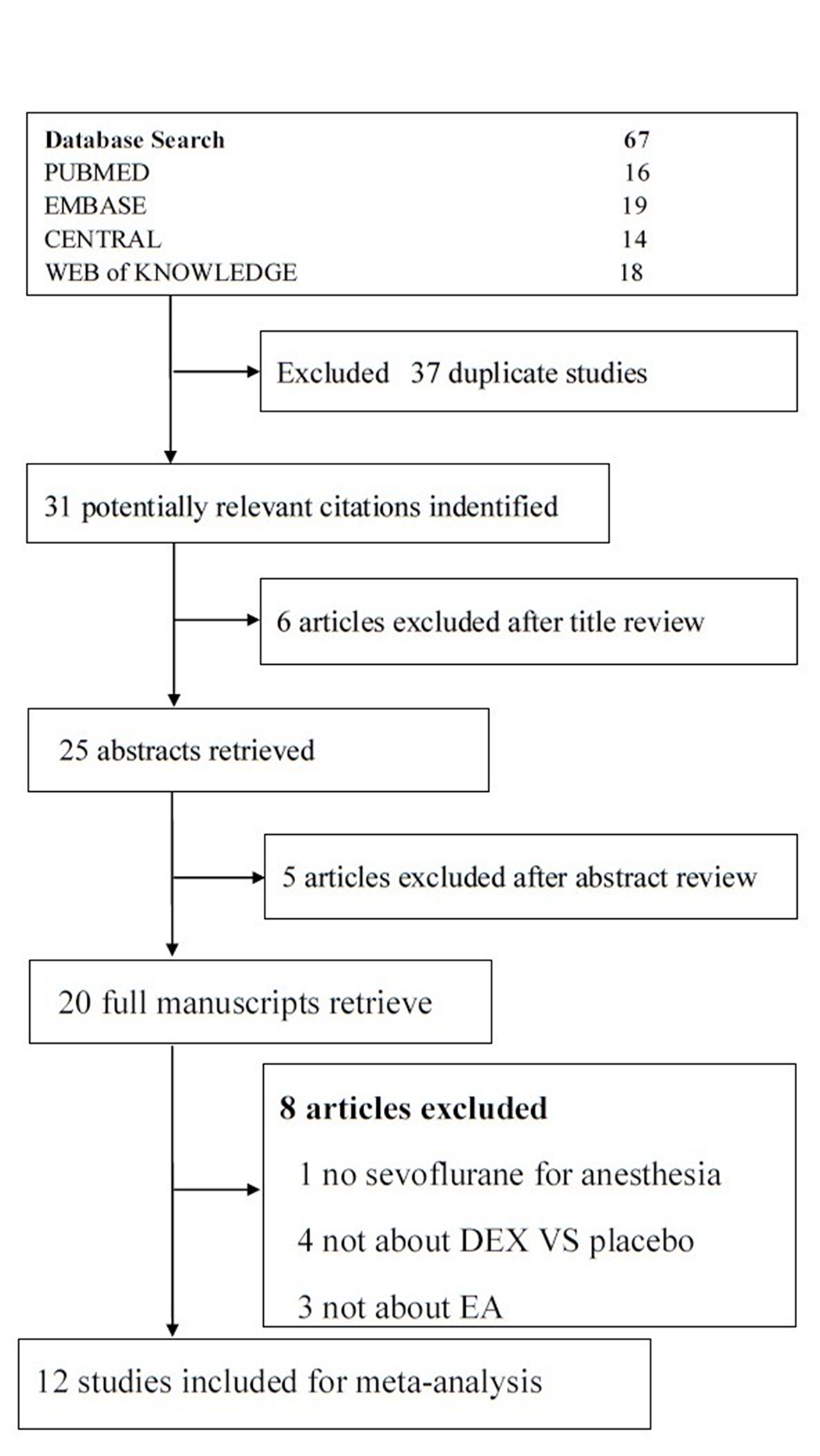 | Literature Search Findings |
| Study characteristics | 18 | Dexmedetomidine was administered by single dose in 9 trials, continuous infusion in 3 trials. The placebo included saline in 11 trials and lactated Ringer’s solution in 1 trial. There were 2 different dexmedetomidine doses examined in 3 trials. For trials that comparison between control group and multiple intervention groups using different dexmedetomidine dose, we combined intervention groups to create a single pair-wise comparison. For dichotomous outcomes, both the sample sizes and the numbers of people with events were summed across groups. For continuous outcomes, means and standard deviations were combined using a formula the handbook recommending. The characteristics of included articles are listed listed below.   | Author  Year | Age( years) | Surgery | Study/Control | Study Intervention | Pre-medication | Sevoflurane (anesthesia) | | Assessment Methods of EA | | --- | --- | --- | --- | --- | --- | --- | --- | --- | | Induction | Maintain | | Ibacache[10]  2004 | 1–10 | Inguinal hernia repair, orchiopexy, or circumcision | 60/30 | Single dose dexmedetomidine 0.15 ug/kg (0.3 ug/kg)IV | No | 8% sevoflurane and 50% N_2_O in O_2_ | 3% sevoflurane in 50% N_2_O | 4-point EA scale > 2 | | Shukry[24]  2005 | 1–10 | Outpatient surgical procedures | 23/23 | Dexmedetomidine in a concentration of 0.2 ug/(kg*h) IV | No | 8% sevoflurane  in O_2_ | sevoflurane to achieve a BIS 40–60 | 4-point EA scale > 2 | | Guler[11]  2005 | 3~7 | Adenotonsillectomy | 30/30 | Dexmedetomidine 0.5 ug/kg IV before the end of the surgery | Acetaminophen15 mg/kg (oral) | 8% sevoflurane and 50% N_2_O in O_2_ | 1.5–2% sevoflurane in 60% N_2_O and 40% O_2_ | 5-point Behavior Scale >3 | | Isik[9]  2006 | 1.5­10 | MRI examination (LMA) | 21/21 | Dexmedetomidine 1ug/kg IV over 2 min after induction | No | 8% sevoflurane  in 2.5 L/min N_2_O and 2.5 L/min O_2_ | 1.5% sevoflurane in 2 L/min N_2_O and 2 L/min O_2_ | 5-point Behavior scale  Of > 3 | | Erdil[18]  2009 | 2-7 | Adenoidectomy | 30/30 | Dexmedetomidine 0.5 mg/kg IV. | 40 mg/kg paracetamol (rectally) | 50% N_2_O and 8% sevoflurane in O_2_ | sevoflurane 1.5 to 2.5% (inspired concentration) in 70% N_2_O/O_2_ | 5-point Behavior scale  Of > 3 | | Sato[21]  2010 | 1–9 | Ambulatory surgery | 39/41 | Dexmedetomidine 0.3 ug/kg IV over 10 min | No | 8% sevoflurane in 6 L/min O_2_ | 2%–5% sevoflurane in 2 L/min O_2_ and 4 L/min air | 4-point EA scale > 2 | | Meng[23]  2012 | 5–14 | Tonsillectomy | 80/40 | Dexmedetomidine 0.5 (1.0) mg/kg IV over10min, maintained with 0.2(0.4) mg/(kg*h) over the surgery | 40 ug/kg midazolam (IV) | None | 1.5%–2.5% sevoflurane fresh O_2_ gas flow of 2.0 L/min | 4-point EA scale > 2 | | Xu[20]  2012 | 3–7 | Vitreoretinal surgery | 30/30 | Dexmedetomidine 0.5 ug/kg IV over a period of 10 min | No | 8% sevoflurane in O_2_ | Sevoflurane (1%–2% end-tidal concentration) in O_2_ | 4-point EA scale > 2 | | Gupta[22]  2013 | 8–12 | Corrective spinal dysraphism | 18/18 | Dexmedetomidine 1 mg/kg bolus over 10 min followed by 0.5 mg/(kg*h) | 0.2 mg glycopyrrolate (intramuscular) | Sevoflurane 8%, | 60% N_2_O in O_2_ and sevoflurane at a fresh gas flow of 3 L/min | 5-point Agitation Cole score > 3 | | Chen[17]  2013 | 2–7 | Strabismus surgery(LMA) | 27/24 | Dexmedetomidine 1 ug/kg IV in the surgery | No | 8% sevoflurane in 5 L/min O_2_ (FiO_2_ = 1.0) | 8% sevoflurane in 5 L/min O_2_ (FiO_2_ = 1.0) | 20-point Pediatric Anesthesia  Emergence Delirium ≥ 10 | | Ali[16]  2013 | 2–6 | Adenotonsillectomy | 40/40 | Dexmedetomidine 0.3 ug/kg IV 5 min before the end of surgery | 0.5 mg/kg midazolam (oral) | 8% sevoflurane and 70% N_2_O in O_2_ | 2%–3% sevoflurane, 60% N_2_O in O_2_ | 5-point Aonos scale > 2 | | He[19]  2013 | 3–7 | Minor surface surgery (LMA) | 61/26 | Dexmedetomidine 0.5 ug/kg (1ug/kg) IV for 10min during surgery | No | 8%sevoflurane in O_2_ | sevoflurane in O_2_ (1 L/min) and air (1 L/min) | 5-point Behavior scale  Of > 3 | | Literature Search Findings and Table S1 |
| Risk of bias within studies | 19 | | Year | study | Random sequence generation | Allocation concealment | Blinding of participants and personnel | Blinding of outcome assessment | Incomplete outcome data | Selective reporting | Other bias | | --- | --- | --- | --- | --- | --- | --- | --- | --- | | 2004 | Ibacache^[1]^ | Low | Unclear | Low | Low | Low | Low | Low | | 2005 | Shukry^[2]^ | Low | Unclear | Low | Low | Unclear | Low | Unclear | | 2005 | Guler^[3]^ | Low | Unclear | Low | Low | Low | Low | Unclear | | 2006 | Isik^[4]^ | Low | Unclear | Low | Low | Low | Low | Low | | 2009 | Erdil^[5]^ | Low | Low | Low | Low | Low | Low | Low | | 2010 | Sato^[6]^ | Low | Unclear | Low | Low | Low | Low | Unclear | | 2012 | Meng^[7]^ | Low | Unclear | Low | Low | Low | Low | Unclear | | 2012 | Xu^[8]^ | Low | Low | Low | Low | Low | Low | Low | | 2013 | Gupta^[9]^ | Low | Unclear | Low | Low | Low | Low | Low | | 2013 | Chen^[10]^ | Low | Unclear | Low | Low | Unclear | Low | Low | | 2013 | Ali^[11]^ | Low | Low | Low | Low | Low | Low | Low | | 2013 | He^[12]^ | Low | Unclear | Low | Low | Low | Low | Low | | TableS2 |
| Results of individual studies | 20 | EA incidence  EA was assessed using a 5-point scale of Agitation Cole score (ACS), Behavior Scale or Pediatric Anesthesia Emergence Delirium (PAED) scale. There were 12 trials that examined the incidence of EA in children under sevoflurane anesthesia. No statistically significant heterogeneity was observed according to the I^2^and Q tests (I^2^ = 0.0%, P = 0.666), and therefore, the fixed effects model was selected. The pooled result showed that dexmedetomidine significantly decreased the incidence of EA in children under sevoflurane anesthesia (RR = 0.346, 95%CI 0.263 to 0.453, P < 0.001，Figure 2). The result was stable when sensitivity analysis was conducted that involved removing 1 trial once from the pooled result (RR_min_ = 0.321, 95%CI_min_ 0.242 to 0.426, and RR_max_ = 0.363, 95%CI_max_ 0.276 to 0.478, Figure 3). The Begg’s funnel plots (P = 0.115) and Egger’s linear regression test (P = 0.110) indicated the probability of publication bias was low (Figure 4).  PONV incidence  PONV mainly focused on the nausea or vomiting which was assessed by behaviors in PACU and for 24hr on the ward. 7 trials examined the incidence of PONV in children under sevoflurane anesthesia. According to the I^2^ and Q tests, there was no statistically significant heterogeneity (I^2^ = 0.0%, P = 0.622), and therefore, the fixed effects model was selected. The pooled result showed that dexmedetomidine significantly decreased the incidence of PONV in children under sevoflurane anesthesia (RR = 0.593, 95%CI 0.391 to 0.901, P = 0.014, Figure 5). However, when the trial of Gupta et al or Chen et al was removed from the pooled trials, a CI of 1 was generated in the 95%CI (0.421 to 1.009 or 0.433 to 1.099 respectively). This decreased the reliability of the test, and therefore, further evidence is required to reach a clear conclusion.  Pain incidence in PACU  Postoperative pain in PACU was assessed by visual analog scale (VAS) or Objective Pain Scale (OPS) during the period in PACU and for 24hr on the ward. There were 5 trials examined the incidence of pain in PACU. Data were homogeneous according to the I^2^ and Q tests (I^2^ = 0.0%, P = 0.879), and therefore, the fixed effects model was selected. The pooled result showed that dexmedetomidine significantly decreased the incidence of pain in children in PACU. (RR = 0.405, 95%CI 0.253 to 0.649, P < 0.001, Figure 6). Removal of individual trials did not significantly alter the result. Funnel plots did not display significant asymmetry.  Extubation time  Extubation time which was measured as the time interval between anesthetic discontinuation and extubation was examined in 9 trials [9,11,16-18,20,22-24]. Data were homogeneous (I^2^ = 31.3%, P = 0.168). The combined result from the fixed effects model suggested that dexmedetomidine prolonged extubation time (WMD = 0.617 min, 95%CI 0.276 to 0.958, P < 0.001, Figure 7). Sensitivity analysis was conducted to examine the influence of each trial on the overall risk estimate and the results were stable.  PACU length of stay  PACU length of stay was examined in 3 trials. We selected the fixed effects model to pool data because data was homogeneous (I^2^ = 0.0%, P = 0.898). We found that PACU length of stay in the dexmedetomidine group was prolonged compared to that in the placebo group (WMD = 4.597 min, 95%CI -0.080 to 9.275, P = 0.054, Figure 8). Sensitivity analysis revealed that the results were stable when trials were removed one by one.  Emergence time  Emergence time was defined as the time from discontinuation of the anesthetic to opening of eyes and was examined in 8 trials. The I^2^ test and Q tests showed that data was homogeneous (I^2^ =0.0%, P = 0.574), and therefore, the fixed effect model was selected. The pooled result demonstrated that dexmedetomidine prolonged emergence time (WMD = 0.977 min, 95%CI 0.392 to 1.561, P = 0.001, Figure 9). Sensitivity analysis showed that the pooled result was not influenced by individual trials.  Adverse effects  There were no serious adverse events such as oxygen desaturation, hypotension, bradycardia, or postoperative respiratory depression in any patient at any time during the study period, except 3children had bronchospasm in control group. | EA incidence, PONV incidence, Pain incidence in PACU, Extubation time, PACU length of stay, Emergence time |
| Synthesis of results | 21 | EA incidence  EA was assessed using a 5-point scale of Agitation Cole score (ACS), Behavior Scale or Pediatric Anesthesia Emergence Delirium (PAED) scale. There were 12 trials that examined the incidence of EA in children under sevoflurane anesthesia. No statistically significant heterogeneity was observed according to the I^2^and Q tests (I^2^ = 0.0%, P = 0.666), and therefore, the fixed effects model was selected. The pooled result showed that dexmedetomidine significantly decreased the incidence of EA in children under sevoflurane anesthesia (RR = 0.346, 95%CI 0.263 to 0.453, P < 0.001，Figure 2). The result was stable when sensitivity analysis was conducted that involved removing 1 trial once from the pooled result (RR_min_ = 0.321, 95%CI_min_ 0.242 to 0.426, and RR_max_ = 0.363, 95%CI_max_ 0.276 to 0.478, Figure 3). The Begg’s funnel plots (P = 0.115) and Egger’s linear regression test (P = 0.110) indicated the probability of publication bias was low (Figure 4).  PONV incidence  PONV mainly focused on the nausea or vomiting which was assessed by behaviors in PACU and for 24hr on the ward. 7 trials examined the incidence of PONV in children under sevoflurane anesthesia. According to the I^2^ and Q tests, there was no statistically significant heterogeneity (I^2^ = 0.0%, P = 0.622), and therefore, the fixed effects model was selected. The pooled result showed that dexmedetomidine significantly decreased the incidence of PONV in children under sevoflurane anesthesia (RR = 0.593, 95%CI 0.391 to 0.901, P = 0.014, Figure 5). However, when the trial of Gupta et al or Chen et al was removed from the pooled trials, a CI of 1 was generated in the 95%CI (0.421 to 1.009 or 0.433 to 1.099 respectively). This decreased the reliability of the test, and therefore, further evidence is required to reach a clear conclusion.  Pain incidence in PACU  Postoperative pain in PACU was assessed by visual analog scale (VAS) or Objective Pain Scale (OPS) during the period in PACU and for 24hr on the ward. There were 5 trials examined the incidence of pain in PACU. Data were homogeneous according to the I^2^ and Q tests (I^2^ = 0.0%, P = 0.879), and therefore, the fixed effects model was selected. The pooled result showed that dexmedetomidine significantly decreased the incidence of pain in children in PACU. (RR = 0.405, 95%CI 0.253 to 0.649, P < 0.001, Figure 6). Removal of individual trials did not significantly alter the result. Funnel plots did not display significant asymmetry.  Extubation time  Extubation time which was measured as the time interval between anesthetic discontinuation and extubation was examined in 9 trials. Data were homogeneous (I^2^ = 31.3%, P = 0.168). The combined result from the fixed effects model suggested that dexmedetomidine prolonged extubation time (WMD = 0.617 min, 95%CI 0.276 to 0.958, P < 0.001, Figure 7). Sensitivity analysis was conducted to examine the influence of each trial on the overall risk estimate and the results were stable.  PACU length of stay  PACU length of stay was examined in 3 trials.. We selected the fixed effects model to pool data because data was homogeneous (I^2^ = 0.0%, P = 0.898). We found that PACU length of stay in the dexmedetomidine group was prolonged compared to that in the placebo group (WMD = 4.597 min, 95%CI -0.080 to 9.275, P = 0.054, Figure 8). Sensitivity analysis revealed that the results were stable when trials were removed one by one.  Emergence time  Emergence time was defined as the time from discontinuation of the anesthetic to opening of eyes and was examined in 8 trials. The I^2^ test and Q tests showed that data was homogeneous (I^2^ =0.0%, P = 0.574), and therefore, the fixed effect model was selected. The pooled result demonstrated that dexmedetomidine prolonged emergence time (WMD = 0.977 min, 95%CI 0.392 to 1.561, P = 0.001, Figure 9). Sensitivity analysis showed that the pooled result was not influenced by individual trials.  Adverse effects  There were no serious adverse events such as oxygen desaturation, hypotension, bradycardia, or postoperative respiratory depression in any patient at any time during the study period, except 3children had bronchospasm in control group. | EA incidence, PONV incidence, Pain incidence in PACU, Extubation time, PACU length of stay, Emergence time |
| Risk of bias across studies | 22 | Publication bias was assessed by Begg's funnel plots and Egger's linear regression test. The results we found suggest all the comparisons did not have statistically significant publication bias. At the same time, the Begg's funnel plots were basic symmetry.  We did not find any obvious bias in the included studies. | EA incidence, PONV incidence, Pain incidence in PACU, Extubation time, PACU length of stay, Emergence time |
| Additional analysis | 23 | Sensitivity analysis was conducted by removing each study individually to assess the quality and consistency of the results. |  |
| **DISCUSSION** | | |  |
| Summary of evidence | 24 | The early stages of EA in children are characterized by crying, excitation, agitation, and delirium. Sevoflurane is associated with a high incidence of EA, and there is a general agreement amongst anesthetists that sevoflurane can increase the incidence of EA in the recovery stage in children compared to propofol. Meta-analysis confirmed that EA occurs more frequently in children under sevoflurane anesthesia than under propofol anesthesia. In addition, another meta-analysis demonstrated that EA occurs more frequently under sevoflurane anesthesia than under halothane anesthesia. The reported incidence of EA following sevoflurane anesthesia varies from 10%–80%. The etiology of EA includes rapid awakening, pain, preoperative anxiety, personality, surgery type, and anesthetic. Furthermore, children between the age of 2 and 5 years are more likely to suffer from EA. EA has additional complications in pediatric patients that include an increased risk of self-injury, dissatisfaction, and associated extra medical care.  A previous meta-analysis showed that the α2-adrenoceptor agonists dexmedetomidine and clonidine were effective in preventing EA related to sevoflurane and desflurane in children. It is difficult to clear which is more effective. So, we only focused on the effects of a single agent, dexmedetomidine to prevent EA in children under sevoflurane anethesia to specifically examine the efficacy of dexmedetomidine. Our meta-analysis suggests that dexmedetomidine can significantly reduce the incidence of EA after emergence from sevoflurane anesthesia in pediatric patients. These results also support dexmedetomidine as an effective and safe agent in preventing EA.  Some authors insist that rapid awakening is the cause of EA. The low blood–gas solubility and rapid recovery characteristics of sevoflurane may contribute to EA. In a meta-analysis of Kanaya et al that showed EA under sevoflurane anesthesia is higher than that under propofol anesthesia in children, extubation time in propofol group was slightly longer (WMD = 1.09 min, 95% CI 0.096 to 2.09), however, because of the significant data heterogeneity, it difficult to confirm rapid emergence plays a role in the higher incidence of EA after sevoflurane anesthesia. In our findings that children administered dexmedetomidine had slightly prolonged extubation time, and emergence time (WMD = 0.617 min, 95% CI 0.276 to 0.958, and WMD = 0.997 min, 95% CI 0.392 to 1.561 respectively), and lower incidence of EA. However, the prolonged time is slight without clinically significant. So, it is difficult to confirm that rapid emergence is a contributing factor to EA.  Pain is considered to be one of the major causes of EA. However, symptoms of screaming, irritability, and anxiety potentially associated with pain are very difficult to distinguish from those of EA, especially in young children. Some studies suggest that EA can be provoked without pain. Isik et al [9] reported that EA was seen in 48% of pediatric patients under sevoflurane anesthesia while undergoing magnetic resonance imaging. Several studies demonstrate that children under propofol anethesia, which does not have analgesia effects, had lower incidence of EA. In addition, children recovered smoothly and pleasantly compared with those under sevoflurane anethesia. Others argue that using fentanyl as a preemptive analgesic can reduce the incidence of EA without delaying emergence associated with desflurane or sevoflurane anesthesia in children. From our meta-analysis, children administered dexmedetomidine had lower incidence of EA, with a low frequency of postoperative pain. Thus, we believe that pain may play a role in the incidence of EA in children.  Dexmedetomidine, a highly specific a2-adrenoceptor agonist with sedative, analgesic, and anxiolytic properties without significant respiratory depression at clinical dosages, has been widely used in pediatric and adult populations. Our findings support several prospective clinical trials in children that dexmedetomidine significantly reduces the incidence of EA after sevoflurane anesthesia. In addition, we found that dexmedetomidine prolonged emergence time and extubation time. Dexmedetomidine is generally well tolerated with few adverse effects. It has little effect on direct memory impairment, respiratory depression, opioid-related pruritus, and PONV at clinical doses. Numerous studies demonstrate that dexmedetomidine has an opioid-sparing effect which can contribute to sufficient analgesia duration, emergence stage, and improve appropriate sedation to offset rapid elimination. The combined actions of attenuated pain and prolonged sedative duration and depth also reduce the incidence of EA. Dexmedetomidine infusions are generally well tolerated with few adverse effects. In all the included trials, we did not find any serious adverse effects. We propose that the sedative and analgesic properties of dexmedetomidine work together to reduce the incidence of EA. Thus, dexmedetomidine appears to be a promising agent to prevent EA in children under sevoflurane anesthesia. | Discussion |
| Limitations | 25 | Our meta-analysis has a number of limitations. First, each study was based on a different study protocol (including the administration methods of dexmedetomidine and sevoflurane) that may cause significant data heterogeneity. However, based on our data analysis at least, we did not find significant heterogeneity. Second, the age range of children differed between the trials examined, with the symptoms of EA being more likely from 2 to 5 years. In our study, age ranged from 1.5 to 14 years, and this large range may have influenced the incidence of EA. | Discussion |
| Conclusions | 26 | Our meta-analysis demonstrated that dexmedetomidine decreases the incidence of EA in children under sevoflurane anesthesia. Our analysis also indicated that dexmedetomidine can decrease the incidence of postoperative pain, prolong emergence time, and extubation time. These findings are reinforced by our sensitivity and publication bias analysis. However, more studies are required to evaluate the effect of dexmedetomidine on the prevention of PONV and PACU length of stay. We propose that dexmedetomidine is a promising agent to prevent EA in children under sevoflurane anesthesia. | Conclusions |
| **FUNDING** | | |  |
| Funding | 27 | No funding for this meta-analysis |  |
